# Imaging intracellular and systemic *in vivo* gold nanoparticles to enhance radiotherapy

**DOI:** 10.1259/bjr.20150170

**Published:** 2015-07-23

**Authors:** S W Botchway, J A Coulter, F J Currell

**Affiliations:** ^1^ Science and Technology Facility Council, Research Complex at Harwell, CLF, Rutherford Appleton Laboratory, Harwell Oxford, Didcot, UK; ^2^ School of Pharmacy, McClay Research Centre, Queen's University Belfast, Belfast, UK; ^3^ School of Mathematics and Physics, Queens University Belfast, Belfast, UK

## Abstract

Nanoparticles offer alternative options in cancer therapy both as drug delivery carriers and as direct therapeutic agents for cancer cell inactivation. More recently, gold nanoparticles (AuNPs) have emerged as promising radiosensitizers achieving significantly elevated radiation dose enhancement factors when irradiated with both kilo-electron-volt and mega-electron-volt X-rays. Use of AuNPs in radiobiology is now being intensely driven by the desire to achieve precise energy deposition in tumours. As a consequence, there is a growing demand for efficient and simple techniques for detection, imaging and characterization of AuNPs in both biological and tumour samples. Spatially accurate imaging on the nanoscale poses a serious challenge requiring high- or super-resolution imaging techniques. In this mini review, we discuss the challenges in using AuNPs as radiosensitizers as well as various current and novel imaging techniques designed to validate the uptake, distribution and localization in mammalian cells. In our own work, we have used multiphoton excited plasmon resonance imaging to map the AuNP intracellular distribution. The benefits and limitations of this approach will also be discussed in some detail. In some cases, the same “excitation” mechanism as is used in an imaging modality can be harnessed to make it also a part of therapy modality (*e.g.* phototherapy)—such examples are discussed in passing as extensions to the imaging modality concerned.

## INTRODUCTION

The use of gold nanoparticles (AuNPs) dates back to ancient Roman times to stain figurines and glasses for decorative purposes. The first known pure colloidal gold (Au) (suspension of submicrometre-sized particles of Au) was from the work by Faraday^1^ in the 1850s. Here, we refer to nanoparticles (NPs) as those with size ≤100 nm. More recently, focus has turned to the therapeutic applications of these materials.

Refined production of existing materials on the nanoscale has driven the nanomedicine revolution for both diagnostic and therapeutic applications.^2^ AuNPs have been used in a variety of ways, including as a drug delivery system designed to optimize the biodistribution of their cargo in cells, tissues and organs.^3–5^ These have proven particularly effective for unstable molecules subject to enzymatic degradation such as DNA, proteins and small-interference RNA oligonucleotides. AuNPs have also improved systemic delivery to hard to reach organs such as the brain.^6^ NP uptake and localization is dependent on size, shape, surface chemistry and the addition of secondary functional groups.^7^ In this regard, target cell type and tissue are critical.

Functionalization of AuNPs confers additional levels of targeting complexity to the NP. These targeting motifs can be used to isolate specific cell populations, thereby providing a means of systemic targeting. For example, specific cell surface proteins such as transferrin are more commonly overexpressed on many tumour cells.^8^ This transferrin-targeting strategy mimics the selectivity of many chemotherapy agents that preferentially target cells with a high proliferative capacity over quiescent or slowly dividing normal cells. Furthermore, high transferrin expression is particularly associated with a malignant phenotype in tumours resistant to conventional therapeutics such as human pancreatic cancer;^9^ therefore, transferrin–antibody conjugated AuNPs could provide novel alternative treatment options.

A key goal of radiotherapy is to increase the radiation dose deposited in the target tissue while minimizing the dose to the surrounding healthy tissue. Progress has been realized through delivery methods involving fractionated dose and the use of multiple beams delivered in various planes thus permitting dose accumulation in the target volume.^10^ Despite these advances, there remains a need to develop new methods, perhaps through the development of molecular targeted radiosensitizing agents, to further minimize non-target radiation damage while simultaneously reducing the risk of secondary cancer development in the normal tissue.

There are a plethora of NP cores under development for health and life science applications. A keywords search in the published literature database (Web of Science) shows that about 50% of this activity concerns AuNPs. It is noteworthy that a larger proportion is found when considering functionalized NPs, reflecting AuNPs' ease of functionalization with the aim of manipulating their uptake and localization. This highlights the importance of being able to accurately determine NP localization using reproducible imaging approaches.

**Figure 1.**
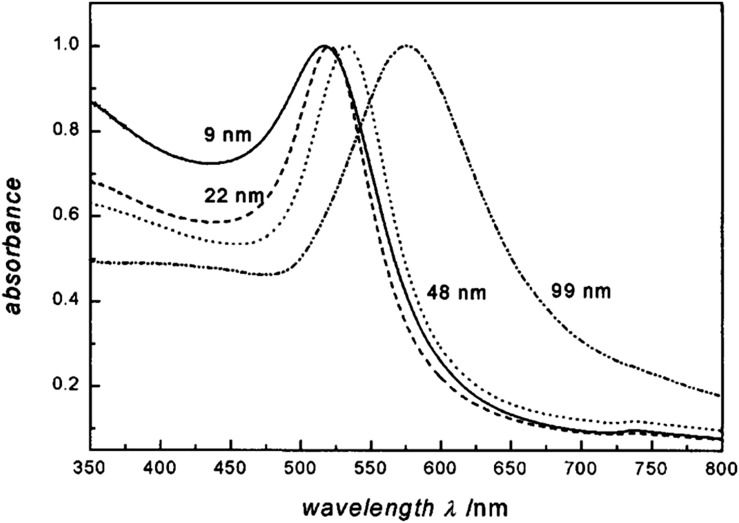
Absorption spectra of 9-, 22-, 48- and 99-nm gold nanoparticles demonstrating a change in surface plasmon resonance with particle diameter. Reproduced from Link and El-Sayed with permission from American Chemical Society.^17^

Tumour-targeted AuNPs may also offer some advantages of heavy ion therapy, by utilizing widely available hospital linear accelerators.^4^ The idea of an increased dose in the presence of high-Z materials is not new and was first proposed in 1949.^11^ High-Z materials such as gadolinium and iodine have been used as contrast agents for decades with several studies reporting that these agents are capable of *in vitro* and *in vivo* damage when used in conjunction with radiation.^12–15^ Being a high-Z material (Z_Au_ = 79), AuNPs are obvious candidates as potential contrast agents. This application is particularly relevant if targeting efficacy is demonstrated, thereby limiting the cost implications whilst improving the expected therapeutic outcome by improving target specificity. AuNPs can be synthesized in the laboratory by a variety of different methods, but most commonly, synthesis involves the reduction of Au salt (chloroauric acid) in the presence of a stabilizer.^16^ The versatility of AuNPs with respect to size, shape, concentration, surface charge and functional groups, combined with the physical properties of Au mean that these agents can be tailored to meet virtually any need in terms of imaging and therapeutic application Figure 1.^18,19^

Furthermore, exploiting the increased mass absorption co-efficient of Au with the aim of enhancing radiation damage through the production of secondary electrons and free radical species has yielded impressive radiosensitization enhancement factors^12^ using relatively low AuNP concentrations. Jain et al^20^ demonstrated this using a commercial thiol-capped 2-nm AuNP at 0.05% wt/wt Au concentration. Under these conditions, dose enhancement ratios of 1.41 and 1.29 were achieved using 160-kVp and 6-MV X-rays, respectively, findings that were supported by other “first generation” NP studies.^21^ This approach has been subsequently optimized through the conjugation of additional ligands designed to confer superior stability and targeting capabilities.

**Figure 2.**
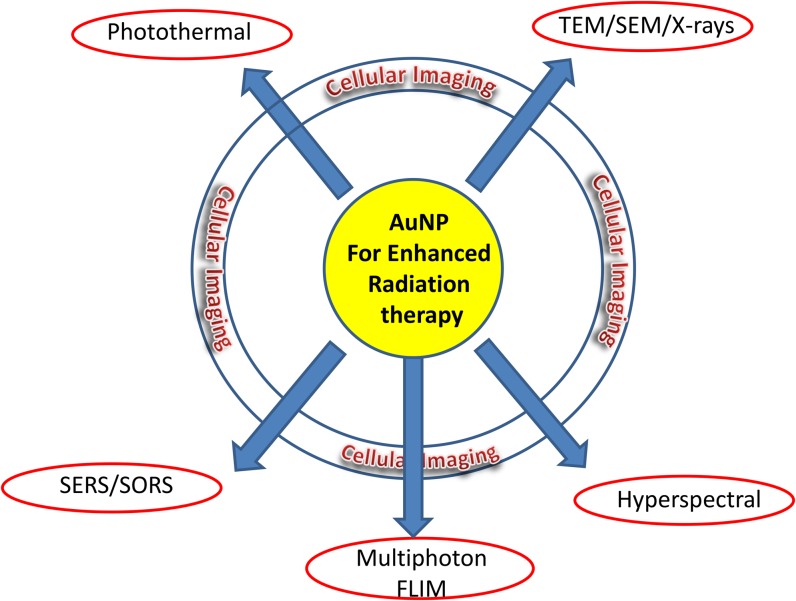
Functional imaging platforms for AuNPs—towards multiply therapeutic methods and multimodality assessment. AuNP, gold nanoparticles; FLIM, fluorescence lifetime imaging microscopy; SEM, scanning electron microscopy; SERS, surface-enhanced Raman spectroscopy; SORS, spatially offset Raman spectroscopy; TEM, transmission electron microscopy.

To date, a major bottleneck in AuNP research relates to effective imaging providing critical information on the kinetics of NP uptake and intracellular distribution (Figure 2).^19^ Several approaches have been developed for this purpose, including the incorporation of a fluorescent tag on the AuNP, thus inferring the subcellular distribution from the corresponding fluorescence signal. However, the ultimate fate of the tag together with the AuNP needs to be confirmed before a firm conclusion may be made. Therefore, this approach is only as secure as the chemical linker attaching the fluorophore tag to the NP. In addition, conjugation of the fluorophore may sterically hinder interaction of functional groups with target receptors or simply alter the intracellular trafficking of the NP, therefore providing an unrepresentative picture of intracellular localization. AuNPs are typically endocytosed by non-specific receptor-mediated endocytosis, resulting in AuNP loaded vesicles fusing with early endosomes, where elevated pH promotes NP agglomeration and detachment of functional groups, ultimately compromising efficacy.^22^ Thus, the fluorescent tag can become detached so that the resultant image does not report on the Au distribution but instead only reports the fluorescent moiety's distribution. Indeed, this is expected to happen as AuNPs are observed to form clusters inside various cell compartments suggesting their coatings are compromised by the cellular chemistry.^7^ Oh et al^22^ showed that AuNPs' cellular uptake was directly dependent on the surface conjugation of a cell-penetrating peptide and that the ultimate intracellular destination was further determined by the diameter of the AuNPs. Also, the smallest (2.4-nm) AuNPs used were found to localize in the nucleus, while intermediate (5.5- and 8.2-nm) particles were largely retained within the cytoplasm, accumulating in relatively high concentration in a perinuclear manner, close to the nuclear membrane.^22^ It is an advantage, and indeed now necessary in clinical treatments, to apply two or more biomedical imaging techniques (multimodality imaging), therefore improving the reliability of diagnostics and treatments, since each imaging technique exhibits its own specific advantages and limitations. For example, detection sensitivities, speed, cost and spatial resolution are all significant issues that should be considered.

In this review, we will detail a number of existing and novel imaging techniques for AuNPs highlighting both strengths and limitations of such approaches, Figure 2.

## SOLUTION PHASE GOLD NANOPARTICLES CHARACTERIZATION

AuNPs may be synthesized by a number of methods, including citrate reduction of Au[III] derivatives such as aurochloric acid (HAuCl_4_) in water to Au(0) and the Brust–Schiffrin synthesis and stabilization by thiols.^23,24^ Recently, a number of one-pot syntheses have been reported for sub 3-nm particle sizes *via* thio-based multidentate fullerene adducts.^25^ The documented cytotoxicity of AuNPs (at certain sizes) means new methods of rendering them safe for cancer therapy is urgently needed. Various approaches developed to achieve this end include AuNP encapsulation in other biologically safe and compatible materials, such as porous Au nanocups and nanospheres.^26^ Hybrid metal oxide such as copper or iron oxide AuNPs with core-shell structure have also been proposed.^27^ Even a graphene-based AuNP hybrid has been developed.^28^ The size of AuNPs produced by conventional techniques may be further tuned or controlled from a few nanometres to tens of nanometres by femtosecond laser fragmentation in water.^29^ Although the synthesis and use of AuNPs for electrochemical applications and as novel sensors for disease states is becoming a significant field, it will not be discussed in this review since it is beyond the scope of this article. However, it is worth highlighting the advances currently being made in this research area.^30^

A number of well-established techniques exist for the physical characterization of AuNPs in solution. Dynamic light scattering (DLS) or photon correlation spectroscopy are techniques that are capable of detailing particle size and are routinely used for calculating the hydrodynamic size of NPs and colloids in solution phase down to 1 nm.^31^ Furthermore, the surface plasmon resonance (SPR) of AuNPs (see the Raman microspectroscopy section) provides a powerful tool for AuNPs conjugated to biomaterials as well as biomolecular binding studies. DLS is able to directly and quantitatively measure the binding stoichiometry between a DNA- or protein-conjugated AuNP probe and a target analyte protein in solution.^32,33^ Although this is not an imaging technique, it is worth mentioning in the context of the broad methods for AuNPs research. However, despite the extensive use of DLS for nanometer size determination, it has specific disadvantages when used with AuNPs. For example, it has been reported that rotational diffusion in particles >30–40 nm, which lack spherical geometries, produces strong scattering effects yielding a false peak in a size range of about 5–10 nm. In this case, the uncritical application of the DLS method may yield particle volume or number size distributions different from those obtained by other methods such as transmission electron microscopy (see transmission and scanning electron microscopes section). The impact of particle size is also evident with smaller diameter particles (<20 nm) that occasionally lead to the observation of peaks at larger sizes, an artefact of particle agglomeration rather than individual particles.^34^ Furthermore, the sensitivity of DLS to AuNP size determination is not consistent from instrument to instrument. The principle of DLS is based on the determination of the autocorrelation function for fluctuations of scattered light intensities. So far there are no reports using DLS in the imaging mode, which limits the technique to only solution studies.

## TRANSMISSION AND SCANNING ELECTRON MICROSCOPES

Owing to the nanometre resolution offered by transmission electron microscope (TEM) and scanning electron microscope (SEM), straight forward visualization of metallic NPs down to a few nanometres should be possible almost without the requirement for any special preparation step. The high electron density of Au means it is again ideally suited for electron microscopy imaging. Indeed, several reports have used TEM and SEM as the main characterization methods for AuNPs following synthesis.^35^ Using these techniques, the size and shape of AuNP down to 1 nm may be observed. By extension, X-ray absorption and reflectivity measurements may also be used to image the AuNPs.^36^ Despite the superior nanoscale resolution possible using these techniques, sample preparation is time consuming and perhaps more importantly relatively destructive, requiring the drying of samples on specialized substrates, ultrathin sectioning and finishing using metal coatings used to provided adequate contrast, decrease charging artefacts and reduce microscope-generated radiation damage. Furthermore, the conductive metal coating used is usually composed of a few nanometre size clusters, which share similar physical properties to the AuNPs employed in medical applications. Therefore, the highly invasive sample preparation steps are not considered compatible with the preparation of live mammalian cells or tumour tissues. Hartsuiker et al^37^ have reported a novel sample preparation protocol where the influence of “charging” on the quality of SEM images could be limited by directly depositing the biological cells on a conductive (Au) surface. To date, some approaches have been developed to minimize these complications such as growing the cells on glass pre-coated with a chromium layer or by developing improved conductive holders composed of a silicone substrate coated with copper. With the later, SEM was used to image 5-nm AuNPs penetrating the skin barrier without the need for heavy metal contrast stains.^35^

## RAMAN MICROSPECTROSCOPY OF GOLD NANOPARTICLES

AuNPs absorb and scatter electromagnetic radiation with extremely high efficiency. This strong interaction with light is owing to the conduction of electrons on the metal surface undergoing a collective oscillation upon absorption of a specific wavelength of light. This specific wavelength is related to the size of the NP (Figure 1). The oscillations are known collectively as SPR and can be exploited for gains in spectroscopy, imaging and therapy. Surface-enhanced Raman spectroscopy (SERS) is a method to enhance the normally poor Raman scattering by several orders of magnitude. Generally, in this technique, molecules are adsorbed on a rough metal surface, such as silver (Ag) or Au for Raman scattering enhancement. The mechanism leading to the enhancement is not fully understood, but a factor of 10^6^–10^7^ electromagnetic enhancement is responsible for the increase of Raman scattering, which occurs as the surface plasmon is excited by the incident light and amplifies the electromagnetic field of the metal surface.^38^

AuNPs have been used in SERS owing to their unique physical characteristics that are critically linked to NP size and shape. It should be noted that Ag is a better SERS agent in solution than Au.^39^ However, the broader biocompatibility profile of Au makes it a more attractive choice for most biomedical applications. It has been suggested in several reports that the shape as well as size of the AuNP is significant for its SERS effect,^40,41^ although the size and shape required for the optimum SERS effect is still under discussion. Hong and Li^42^ have investigated a AuNP size range from 17 to 80 nm (nearly spherical in shape) for the SERS effect and concluded that 50 nm was the optimum size for the enhancement together with reduced cellular cytoxicity. This AuNP's dimensions are within the size used in medical applications including radiobiology.

One application of SERS is discrimination between cancer and non-cancer cells with enhanced signal detection, giving it a role in disease detection. SERS benefits from the chemical specificity offered by Raman spectroscopy—one of the reasons is that this form of spectroscopy has been widely applied in biomedical and clinical diagnostic applications. Direct conjugation of AuNPs to biomarkers allows them to act as biospecific nanoantennae partially overcoming the inherently weak signal associated with Raman spectroscopy. The recent development of spatially offset Raman spectroscopy (SORS and its variants) for non-invasive detection of small, deeply buried lesions (up to 6 cm) makes it possible.^43,44^ Matousek et al have recently demonstrated the technique for non-invasive breast cancer detection possibility at depths around 2 cm.^45^

**Figure 3.**
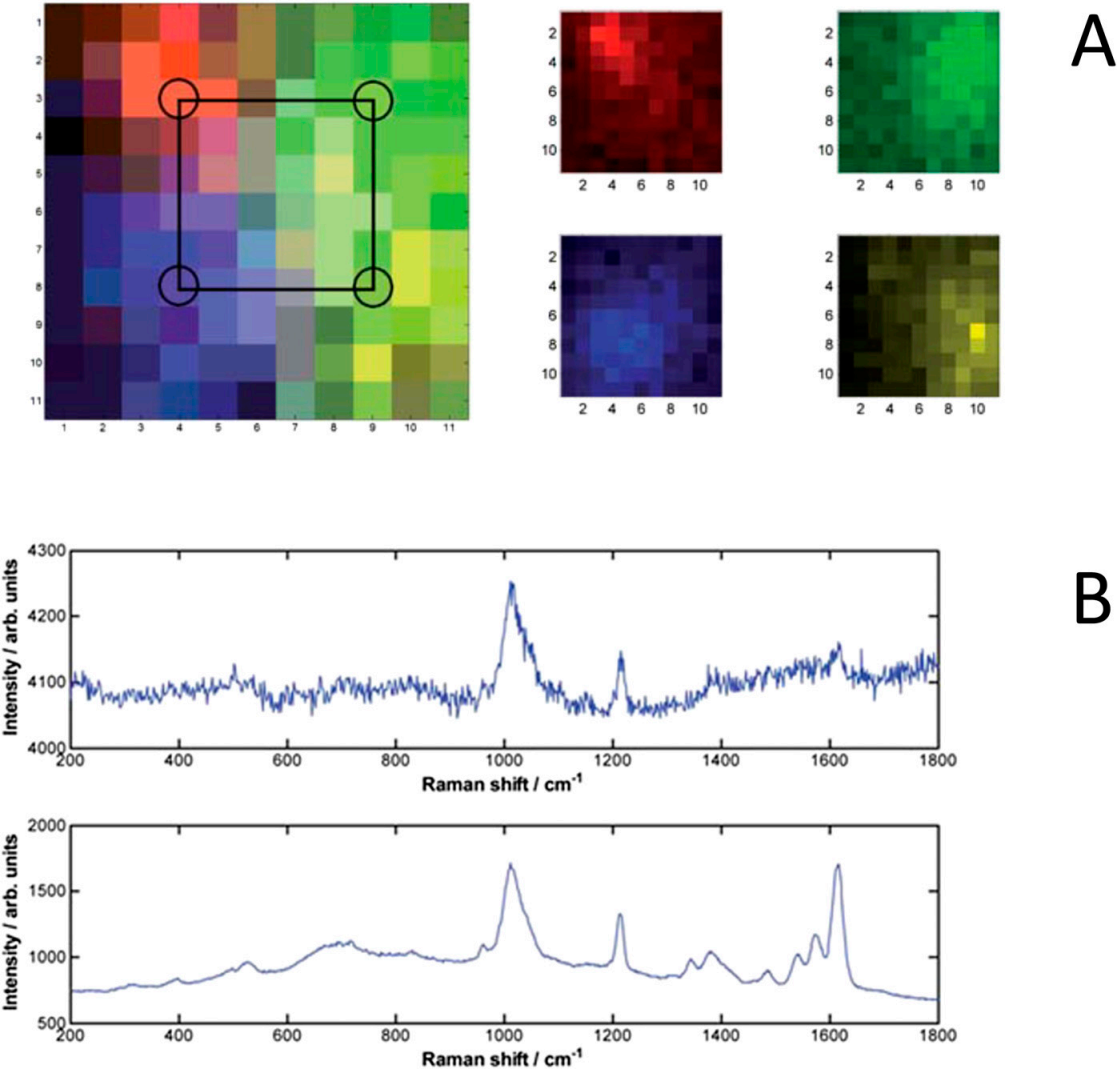
Surface-enhanced Raman spectroscopy (SERS) nanoparticle (NP) signals (a) diffuse scattering of the Raman photons produced by the NPs deep within phantom tissue. False colour images of the SERS NP signals, measured in a 11 × 11 grid, pixel size 2 mm. Left image shows all signals plotted together (with pixel colour mix showing combined signals) with the approximate injection points marked, right image shows each “flavour” separately. Red is ×403, green is ×420, blue is ×421, yellow is ×440. (b) Raw spectra of NP flavour ×403 with 3 × 10^10^ particles in 50 μl for 47 mm (range 45–50 mm) × 50 × 50 mm tissue block (top frame) and 1.8 × 10^9^ particles in 3 μl for 20-mm thick × 50 × 50 mm tissue block (bottom measured in a 11 × 11 grid, pixel size 2 mm through thick tissue material). Reproduced from Stone et al with permission from Royal Society of Chemistry.^45^ For colour image see online.

The combination of SERS from AuNPs and SORS has further improved the prospect for *in vivo*, non-invasive, specific detection of molecular changes associated with disease up to depths of around 5 cm representing a significant improvement over traditionally detected Raman signals by two orders of magnitude (Figure 3).^45^ Therefore, dual detection approaches utilizing the specificity of the Raman spectra signature, combined with the use of functionalized AuNPs for tumour targeting, could provide a strategy for improved definition of tumour boundaries, thus permitting improved radiotherapy delivery to the tumour volume.

Adopting a similar strategy, AuNPs complexed with dextran-coated superparamagnetic iron oxide NPs were established for non-invasive combination *in vivo* imaging, combining MRI and Raman spectroscopy.^46,47^ This multimodal approach offers the combination of different modalities into one system thus compensating for the deficiencies of single utility approaches. The SERS capability in this combination is particularly valuable, since it is highly sensitive and the detailed information gained by this method can readily be distinguished from background tissue signatures.

AuNPs are surrounded with Raman reporters, which provide light emission that is >200 times brighter than quantum dots. It was found that the Raman reporters were stabilized when the NPs were encapsulated with a thiol-modified polyethylene glycol (PEG) coat. The same combined multicomponent AuNP (or nano crystals) has been reported as a nanotheranostic agent for successful bimodal ultrasound/MRI and guided photothermal ablation in human tumour xenograft models^48^ (see the Multiphoton detection and imaging of gold nanoparticles section).

## HYPERSPECTRAL IMAGING

Hyperspectral microscopy allows simultaneous spatial and spectroscopic characterization of non-fluorescent samples. In practice, the technique can be simple and relatively straight forward, requiring only a simple microscopy set-up together with a spectral detection camera with the flexibility of expanding the set-up to include more expensive supercontinuum lasers.^49^ Hyperspectral microscopy involves excitation of the sample with a range of wavelengths (or white light) simultaneously followed by detection of the dispersed spectral component by the sample under investigation, in this case AuNPs. The spectral components may then be used to characterize the AuNP following the dispersion through a prism or by a diffraction grating. In general, the usual two-dimensional (*x*, *y*) image is accompanied by a third component, wavelength, to give an image, that is (*x*, *y*, *λ*). The data set is therefore composed of images representing a narrow wavelength range of the electromagnetic spectrum.

Patskovsky et al^50^ have used hyperspectral dark-field microscopy to detect and image PEGylated AuNPs targeting CD44-expressing cancer cells. A tuneable filter was used to sweep the white light illumination source from a supercontinuum laser over a spectral range from 400 to 1000 nm. Although the advantage of the added dark-field methods to the hyperspectral technique is not immediately obvious, the authors believe that this allowed them to differentiate between AuNPs that were cell bound and those randomly attached to the glass surface.

## PHOTOTHERMAL OPTICAL COHERENCE TOMOGRAPHY AND PHOTOTHERMAL IMAGING AND THERAPY

Gold nanorods (AuNRs) have also been used as a potentially powerful tool for molecular imaging in photothermal optical coherence tomography (PT-OCT).^51,52^ *In vivo* PT-OCT images showed an increase in signal in the presence of AuNRs compared with control samples. Additionally, *in vivo* PT-OCT AuNR signals were spatially distinct from blood vessels imaged with Doppler optical coherence tomography. Similarly, graphene-isolated-Au-nanocrystal nanostructures for multimodal cell imaging and photothermal-enhanced chemotherapy have been proposed.^53^ In the above report, the authors applied a thin layer of graphene on the surfaces of the AuNPs or AuNR crystals. This led to some unique capabilities: (1) the surface-enhanced-Raman-scattering substrate quenches background fluorescence and reduces photocarbonization or photobleaching of analytes; (2) the AuNPS may be used for multimodal cell imaging by both Raman spectroscopy and near-infrared (NIR) two-photon luminescence; (3) the graphene-isolated AuNPs provide a platform for loading anticancer drugs for therapy as discussed above; (4) the NIR absorption properties nano material with graphene gives photothermal therapeutic capability in combination with chemotherapy. Controlled release of chemotherapy molecules from the graphene-isolated AuNPs may be achieved through NIR heating, significantly reducing the possibility of side effects in chemotherapy. The decorated graphene AuNPs have high surface areas and stable thin shells, as well as unique optical and photothermal properties, making them promising nanostructures for biomedical applications and in particular tumour inactivation. Although such exotic AuNPs have not been investigated in conjunction with ionizing radiation, there is no reason why an increased radiobiological effectiveness in cell killing should not be possible. It is interesting to note that radioactive-iodine-labelled AuNRs have been designed and synthesized for target selective single-photon emission CT (SPECT) and X-ray CT imaging and subsequent thermal ablation of folate-receptor-overexpressing cancers *via* photo thermal therapy.^54^ Similarly, patients with localized prostate cancer are often treated with brachytherapy using iodine-125 (^125^I) or palladium-103, which emit X-rays and gamma rays of maximum energy 35 and 21 keV, respectively. Cho et al^55^ specifically modelled ^125^I brachytherapy seeds in tumours loaded with 18 mg Au g^−1^. Thus, a combined localized AuNP radiation and photothermal therapy (PTT) represents a future possibility.

PTT, unlike photodynamic therapy (PDT), does not involve chemical photosensitizers for the generation of reactive oxygen species to produce the targeted killing of cancer tissue. Rather, it requires the passive accumulation of NPs within the tumour mass owing to the poor structural integrity of the tumour vasculature and the subsequent laser excitation of the NPs to generate ablative intracellular temperatures. PTT results in a temperature increase (approximately 10°C) in the environment surrounding the target, leading to thermoelastic expansion of the sample and shifts in the local refraction index.^56,57^ PTT with AuNPs have been investigated as potential candidates in this anticancer strategy.^58^ However, the optimal absorption wavelength of spherical AuNPs with diameters ranging between 10 and 80 nm^42^ is <580 nm, thus falling short of the so-called typical transparent tissue window of 650–1350 nm, where the excitation light source has the maximum ability to penetrate deep (several millimetres) into tissue. This property clearly limits the usefulness of spherical AuNPs in this context. However, altering the geometry of the NPs to produce AuNRs can reduce this effect by tuning the optimal absorption wavelength to 1000 nm. This approach was adopted by Popp et al^59^ who synthesized a PEG coating to reduce the aggregation and suspected toxicity associated with AuNPs of <10 nm. The accumulated AuNPs may then be irradiated with varying radiation energies to create sufficient heat for the cancer cell killing. The PEG stabilized AuNRs for the PTT treatment used a high powered light-emitting diodes or laser excitation source in the treatment of a murine melanoma model. Three key parameters have been highlighted as essential features for PTT: (1) accurate identification of the location and size of tumours together with the presence of nanoscale photoabsorbers for photonic irradiation before therapy; (2) monitoring the treatment procedure in real-time during therapy to ensure complete eradication of microscopic tumour; (3) assessment of the effectiveness of the therapy following the treatment.^48^ Imaging therefore plays a critical role in the monitoring process of PTT. The intrinsic luminescence following PTT may be used to detect the AuNPs.^60^ In general, the PDT drugs are fluorescent and can therefore be used to image the tumour environment whilst new reports are beginning to emerge for the use of PT imaging using the same NPs for PTT.^60^

## MULTIPHOTON DETECTION AND IMAGING OF GOLD NANOPARTICLES

In the Raman Microspectroscopy of Gold Nanoparticles section, we discussed efficient plasmon resonance from AuNPs upon one-photon excitation with visible light. The SPR is known to be wavelength dependent, being blue-shifted for smaller particle size.^61^ This resonance can be excited through two-photon excitation using picosecond and femtosecond laser pulses. Two- and three-photon processes [collectively termed multiphoton (MP) process] have now been reported for AuNPs.^62^ MP absorption, first proposed in 1931 by Göppert-Meyer^63^ and demonstrated with lasers in the 1960s^64^ is now widely employed in MP excited fluorescence microscopy.^65,66^ Two- or three-photon excitation, which have very low cross-sections, most readily occur at a high photon density within the femtolitre focal volume of a subpicosecond pulsed-laser excitation beam, which may then be scanned across a sample to construct a fluorescence image.^67,68^ An important advantage in both biological imaging and phototherapy is that MP excitation shifts the required wavelength to the red or NIR spectral regions where light transmission in human and animal tissues is much greater than at shorter wavelengths.^69^ However, a major concern relates to the depths that may be probed within a sample, which is dependent upon several factors: light penetration and good transmission with low scattering combining to permit the necessary high peak laser power at the focus.^70^ For three-dimensional imaging of intact tissues, the choice of the wavelength is important, and recent work^71^ suggests that wavelengths even longer than the commonly used range of 800–1000 nm may be optimal. For fixed tissues, optical clearing methods have been devised, which substantially increase the sample transparency.^72^ However, at this early stage of utilization of MP imaging of AuNPs, the work primarily centres on cells grown in monolayers; therefore, the issue of light penetration can be ignored in this present review. MP excitation delivery to deep-lying (several millimetres) tumours is also being addressed by microscope manufacturers who are now offering specially designed lenses to optimize deep light penetration in tissues, which combine specifications such as long working distance, silicon oil immersion for refractive index matching, chromatic aberration minimization and high numerical aperture. AuNPs combined MP imaging could therefore offer a novel opportunity to study the outcome of uptake, distribution and radiobiological effectiveness following irradiation by imaging in fixed, live cells and tissues.^73^

The luminescence from AuNPs excited with the MP process overlaps with that from the autofluorescence from cells and tissues (<600 nm). Therefore, a method to differentiate between AuNP fluorescence from cellular autofluorescence is necessary.

**Figure 4.**
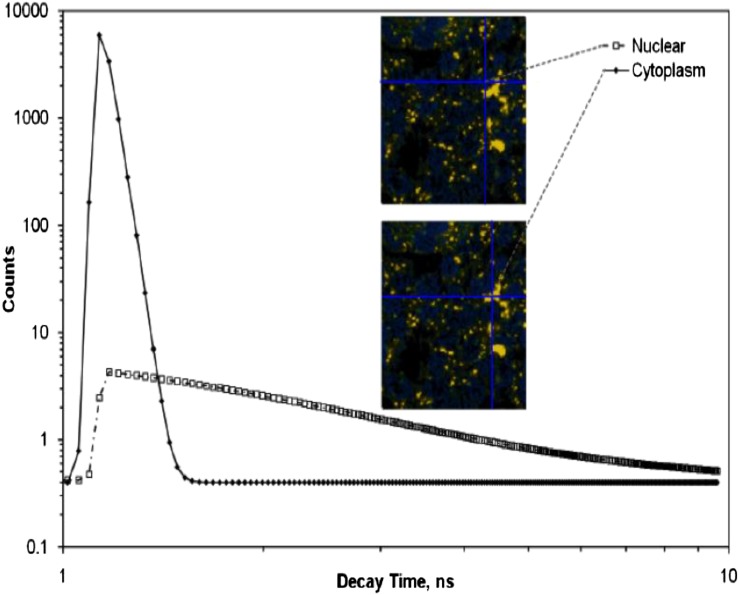
The surface plasmon resonance observed through multiphoton excitation with the laser tuned to 900 nm. Aggregates of gold nanoparticles can be seen with characteristically short decay time, compared with the longer decay time of the cytoplasm. See the Do gold nanoparticles induce cellular DNA damage? section for details.

**Figure 5.**
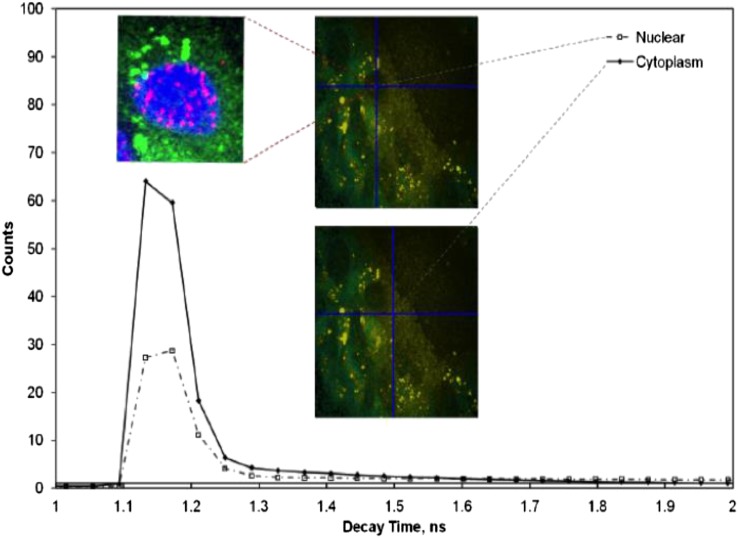
The surface plasmon resonance observed through multiphoton excitation with the laser tuned to 600 nm. Smaller nanoparticle aggregates are now detected both in the cytoplasm and (with much lower concentration) in the nucleus. The insert also shows results from a co-staining experiments where gold nanoparticle location and DNA damage post-irradiation are correlated. See the Do gold nanoparticles induce cellular DNA damage? section for details.

Fluorescence lifetime imaging microscopy (FLIM) has the potential to provide such discrimination. Utilizing confocal, single and MP excited state emission microscopy together with time-correlated single-photon counting provides an unambiguous determination of the location of fluorophores on a pixel-by-pixel basis.^74^ The fluorescence lifetime for a particular molecular environment is exact for every fluorescent molecule and defines the average time a molecule spends in the excited state before returning to the ground state, which is typically in the range of several nanoseconds for fluorescent proteins. The decay of fluorescence does not depend on its concentration (<1 mM) but is sensitive to events in its local microenvironment. When excited with pulsed laser wavelength, the plasmon resonance generated from AuNPs also follows closely the laser pulse length characteristics.^75^ This highlights the requirement for femtosecond lasers for MP excitation, which are superior to using nanosecond or continuous wave laser pulses. Furthermore the MP process has the potential of generating ultrashort plasmon resonance (in femtoseconds) as well as second and third harmonic generations.^76^ Therefore, the time signature of the resultant subsequent decay may be used to distinguish between the prompt decay owing to Au and slower decay processes indicating autofluorescence arising from surrounding biological matter (Figure 4). As is illustrated in Figure 1, the maximum absorbance spectra of AuNPs is dependent on size. This property can be used to discriminate between clumped and unclumped AuNPs in cells since clumps act like a single larger NP. When the excitation laser is tuned to a shorter wavelength, the distribution of smaller clumps and unclumped NPs may also be observed (Figure 5). Varying the excitation wavelength from 570 to 900 nm, we are able to selectively image AuNPs of varying sizes. Larger aggregates are observed with excitation wavelength >700 nm while excitation with ≤600 nm shows less aggregate particles. The exact sizes of AuNPs imaged by the technique at the ≤600 nm are not fully characterized as yet. The small insert in this image above the peak in the temporal profile in Figure 5 shows the power of this technique combined with confocal fluorescence microscopy. Here, a three-colour image is shown with green showing the AuNP density measured by MP FLIM. The blue shows the nuclear DNA and the magenta DNA damage, both measured using confocal microscopy. Measurements of this kind have been used to determine mechanisms of DNA damage enhancement induced by AuNPs upon irradiation [H McQuaid, 2015, personal communication].

**Figure 6.**
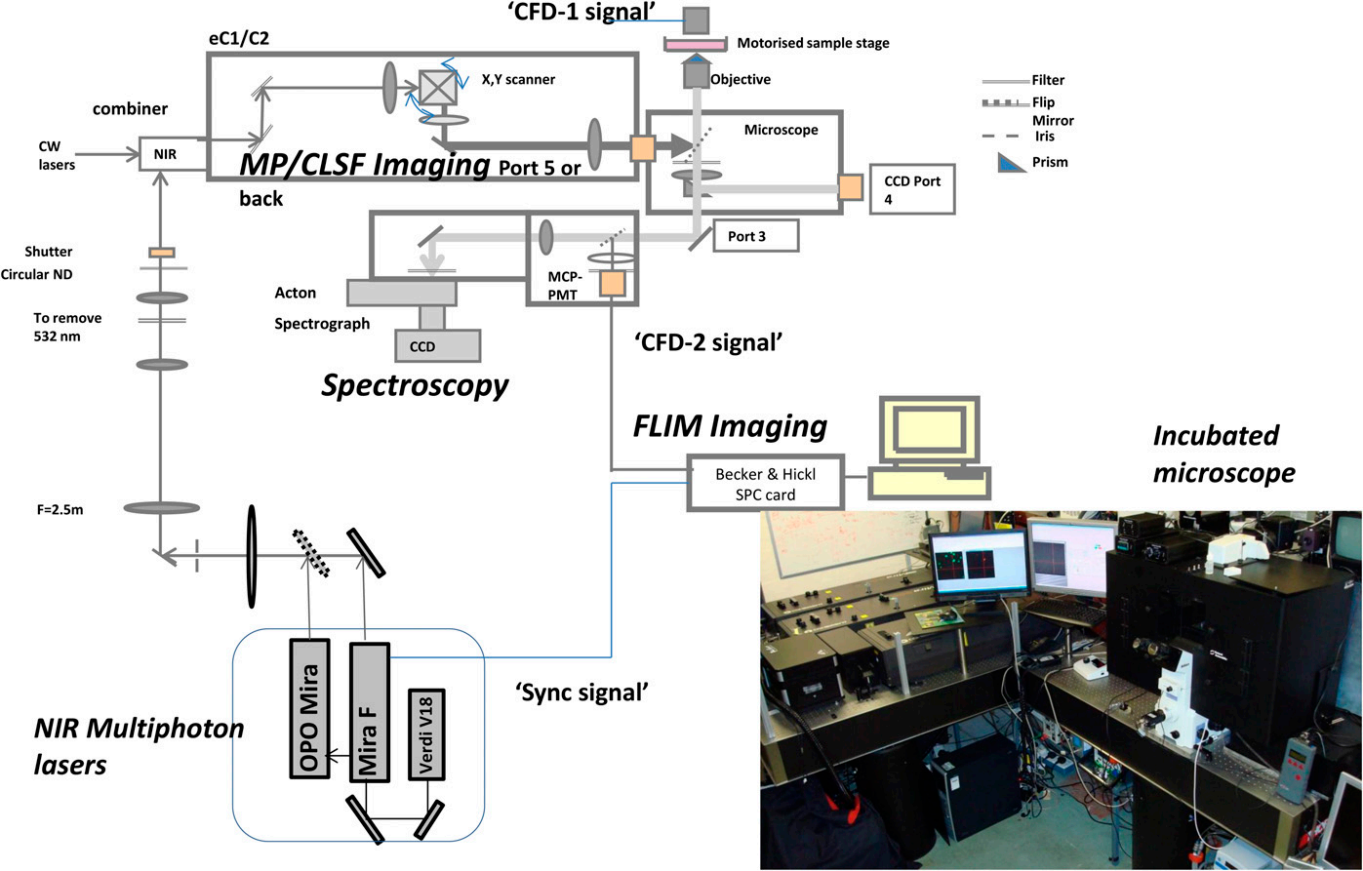
Schematic layouts of the modifications to the standard Nikon confocal microscope for multiphoton (MP) excitation and fluorescence lifetime imaging microscopy (FLIM) to enable plasmon resonance imaging of gold nanoparticles. Reproduced from Botchway et al with permission from John Wiley and Sons.^77^ CCD, charge coupled device; CFD, constant fraction discriminator; CLSF, eclipse (version 1 and 2), confocal laser scanning fluorescence; CW, continuous wave; MCP-PMT, micro-channel plate photomultiplier tube; ND, neutral density; NIR, near-infrared.

**Figure 7.**
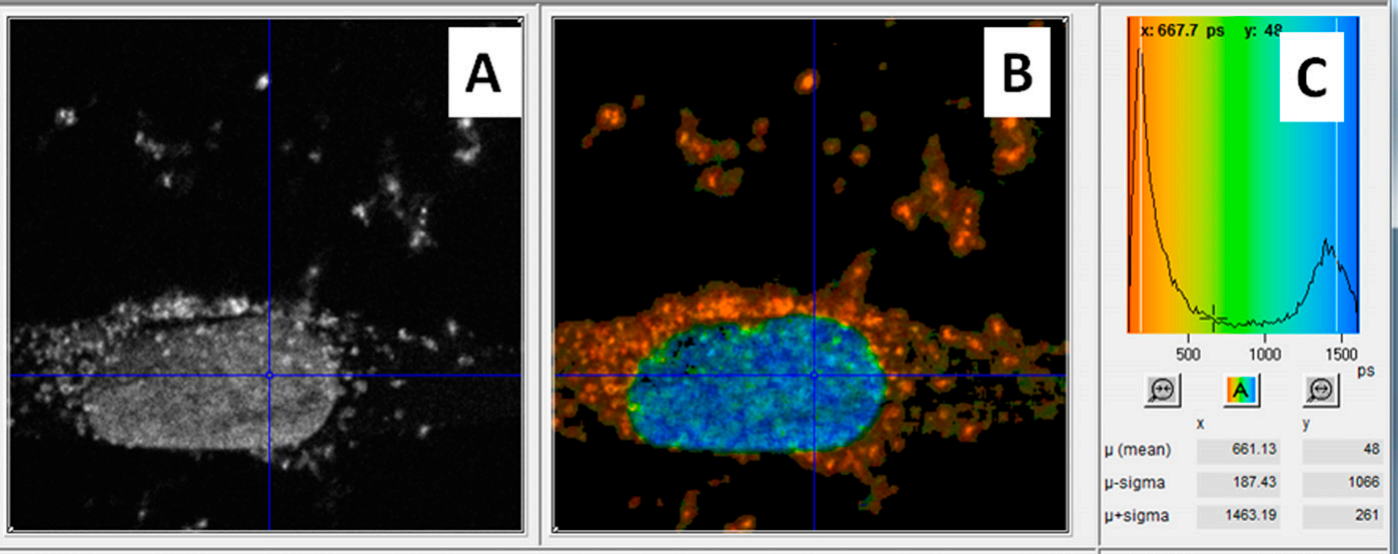
Dual gold nanoparticles and DAPI imaging by multiphoton plasmon resonance FLIM of a single cell. (a) Steady-state fluorescence intensity image. (b) Calculated fluorescence lifetime image at each pixel (256 × 256 pixels). (c) False colour-coded distribution of lifetimes across all pixels in the image.

Images are produced by raster scanning the tightly focused spot of the excitation laser across the sample whilst recording the higher energy photons produced with a fast photomultiplier. The MP-FLIM technique could be built as a standalone unit or constructed around a standard confocal or steady-state MP set-up. The former system provides a more flexible set-up where the detectors are optimized for detection from ultraviolet (UV) to visible.^76^ In the latter set-up, the functionality of a standard confocal microscopy is extended to incorporate MP lasers as well as an optional output for collecting the second harmonic generation and plasmon resonance from the AuNPs following excitation (Figure 6) [H McQuaid, 2015, personal communication]. We have shown using this approach that the fast component of AuNPs' higher energy photon as well as the fluorescence emission from fluorescent probes as labels may be observed simultaneously (Figure 7).

MP laser induced breakdown spectroscopy (LIBS) is a technique (that shares much of the same hardware as MP microscopy) being applied to imaging gadolinium-based NPs and is expected to find application in AuNP imaging. Although the average laser powers used for MP imaging is low enough (<5 mW) not to cause ionization of the NPs, a doubling of the imaging laser power may be sufficient to initiate this LIBS (generation of a plasma plume) and the imaging thereof.^77^ This approach further adds to the non-fluorescent imaging techniques that may be useful for AuNPs research and application.

## DO GOLD NANOPARTICLES INDUCE CELLULAR DNA DAMAGE?

The classical concept of radiation action in tumour and cell killing is by cellular DNA damage and in particular the induction of DNA double-strand breaks that are thought to be the most deleterious. There is still a debate on whether tumour and cellular inactivation by combined X-rays (low linear energy transfer) and in the presence of AuNPs is driven by DNA damaging mechanisms, with various conflicting reports published.^20,78–81^ Much of the early work, investigating the radiosensitizing potential of AuNPs was performed using extrachromosomal plasmid DNA isolated from transformed competent bacteria. These assays provided a sensitive, rapid and low-cost approach for evaluating the DNA damaging potential of exogenous agents, both alone and in combination with radiation. Using this model, Butterworth et al examined the impact of AuNP size, concentration and the scavenging environment on radiosensitization, reporting two-fold AuNP-induced dose enhancement factors for both single- and double-stranded DNA lesions.^81^ However, despite a more detailed understanding of the abundance and limited functional range of the various secondary electron species produced following AuNP/radiation interactions,^12^ the induction of intracellular DNA double-strand break damage remained widely accepted as the obvious mechanism resulting in additional cell death. Within the cellular environment, TEM observations of AuNP entrapment in endosomal/lysosomal compartments were commonly reported, with no apparent nuclear accumulation.^7,81^ Indeed, various images present large NP aggregations micrometres from the nuclear membrane. When considered alongside Monte Carlo calculations, modelling dose distribution on the nanoscale, which indicate 99.9% of the enhanced dose deposited, has a maximum range of 50 nm; it seems unlikely that increased cell killing effects are solely attributable to increased DNA double-strand break induction.^12^ This was apparent when Jain et al^20^ observed no increase in DNA damage yields across three cell lines of different origin, despite reporting clear AuNP-mediated radiosensitization in MDA-MB-231 cells, indicating radiosensitization was driven by alternative mechanisms. Second generation AuNPs, conjugated with functional groups, have been developed to help overcome the issues of endosomal entrapment and to promote nuclear accumulation.^21^ Conjugation of short biomimetic peptides of viral origin have improved AuNP uptake, allowing the use of lower therapeutic concentrations while simultaneously promoting endosomal escape and nuclear targeting.^81,82^ In the later study,^82^ this equated to a five-fold enhancement of uptake relative to unfunctionalized control NPs, along with a two-fold reduction in the exocytotic potential. The impact of effective functional groups was neatly demonstrated when comparing DNA double-strand break induction using human epidermal growth factor receptor-2 targeted AuNPs relative to non-targeted NPs. Radiosensitization and low-level DNA damage induction were observed without conjugation of trastuzumab, equating to a dose enhancement factor of 1.3 and increased γ-H2AX foci yields of 1.7-fold over radiation only. However, the targeting efficacy conferred by the monoclonal antibody increased the radiosensitizing effect by two folds, and the DNA damage yields by 3.28 folds. *In vivo*, the significance of this translated into a 46% regression in tumour volume relative to a 16% increase in radiation-only treated animals over a time-matched period of 118 days.^79^

## GENERAL MATERIALS AND METHODS FOR USE OF GOLD NANOPARTICLES IN CELLS AND MULTIPHOTON IMAGING

For MP plasmon resonance imaging, cells were seeded onto sterile 16-mm^2^ round coverslips placed in six-well plates at a density of 1 × 10^5^ cells per well. Cells were left to propagate for 4–6 h before treatment with 2-nm AuNPs at a concentration of 12 μM for 24 h. Cells were then fixed with a solution of 50% acetone and 50% methanol for 10 min before being washed with phosphate-buffered saline (PBS) and nuclei stained with 4', 6-diamidino-2-phenylindole (DAPI) at a concentration of 20 µg ml^−1^ for 10 min. DAPI was removed and cells were washed in PBS twice before being mounted onto glass microscope slides with 5 µl of VECTASHIELD^®^ Mounting Media (Vector Labs Ltd, London, UK) and sealed with nail varnish.^20^

To detect the AuNPs' localization in cells, the samples were illuminated with tightly focused (<500-nm beam waist) laser pulses of short duration (approximately 200 fs) through a ×60 water immersion objective (NA 1.20), at a wavelength of 580–900 nm. The wavelength (>700 nm) and characteristic pulses were produced using either the titanium–sapphire laser (Mira 900F; Coherent Ltd, Ely, UK) or an optical parametric oscillator (<650 nm) (OPO; APE, Berlin, Germany) pumped by the titanium–sapphire laser. The decay of the SPR resulted in both visible and UV photons, which after passing through an UV bandpass filter (U340; Comar Instruments, Cambridge, UK), was detected using a R3809-U photomultiplier tube (Hamamatsu Photonics, Hamamatsu City, Shizuoka Pref., Japan). The lifetime of the decay was measured using a Becker & Hickl time-correlated single-photon counting system (SPC830) and analysis software (SPCImage v. 3.9; Becker & Hickl GmbH, Berlin, Germany). This process was repeated many times as the laser was raster-scanned across the sample to accumulate the signal depending on the concentration of AuNPs and intensity recorded. For each location, the lifetime spectrum was analysed in terms of a fast and slow component to generate an image of the subcellular AuNPs distribution since the fast component corresponds to the decay of the plasmon resonance of the AuNPs while the slow component arises from non-linear excitation of the cytoplasm or nuclear DNA.

## CONCLUSION

At the time of writing this review, there are no imaging techniques capable of imaging AuNPs <20 nm in size under physiological conditions in cells. All the techniques described here have some advantages and significant disadvantages. However, the MP plasmon resonance combined FLIM is proving to be a powerful approach to imaging AuNPs and the clusters that form upon accumulation in cells. The ability to co-register MP (Au location) images with traditional confocal images is useful for both mechanistic and uptake studies. In this regard, the fate and speciation of AuNPs attached to other ligands may be followed so that (a) the excited state lifetime of a ligand, if longer than the extremely short plasmon resonance, may be used to detect its location *vs* (b) that of the AuNPs if the two separate upon uptake. Future developments should allow these benefits to be extended to shorter times and with more comprehensive dynamical information through the implementation of new data acquisition modes to be used alongside live-cell imaging.

The mechanism of AuNP-induced tumour destruction and cell killing is still being debated as the classical DNA damage, particularly double-strand breaks does not appear to apply. The multimodality imaging and tumour therapy has been highlighted in some reports but not fully explored. It is expected that these combinatorial therapy/imaging approaches described here would gain in popularity and importance once the techniques become better understood.
